# Editorials

**Published:** 1911-02

**Authors:** 


### EDITORIALS

The Business Office of the *Journal-Record* is 1 1-2 to 5 1-2 South Broad Street.
The Editorial Office is 1014-15 Century Building.
Address all Business Communications to *Journal-Record of Medicine*, 1 1-2 to 5 1-2 South Broad Street.
Make remittances payable *to The Journal-Record of Medicine.*
On matters pertaining to the Editorial and Original Communications, address Edgar G. Ballenger, M. D., Atlanta, Ga.
Reprints of Original Articles will be furnished at cost price. Requests for the same should always be made in THE MANUSCRIPT.
We will present, postpaid, on request, to each contributor of an original article, twenty (20) marked copies of *The Journal-Record of Medicine* containing such article.

### SALVARSAN AND THE PROGNOSIS OF SYPHILIS.

Now since salvarsan, or "606," has come into more or less general use, we are all confronted with the important question of how many doses to give, how soon to repeat them and when is the patient cured. While we have in "606" a remedy which cures active syphilitic lesions in a remarkable manner and while we can doubtless effect more cures with it than we have been able to do before, *we have not made a similar advance in determining positively when the patient is entirely well of the disease.* In former communications the writer has urged caution in the ultimate prognosis and he still urges that no patient should be considered cured until he has remained under observation for two or three years, free from the disease and with frequent negative Wasserman reactions. All conservative men admit that, since we know syphilis to be such a treacherous disease, no statement can be made as to the permanency of the cure until the time test has proven it.

Since all reports have shown that some patients have relapses and a few fail to respond at all to "606," we certainly must be fair with the patient, fair to ourselves and fair to the remedy and not make definite promises as to the absolute cure with a single injection. As we have no method of determining which patient may have a relapse, I think it most important to Insist that all patients have the second injection at the end of a month, no matter how excellent the temporary results may appear. If, then, repeated blood tests are negative for many months, and the patient remains free from symptoms, other treatment might be omitted until the time test has proven the patient to be well. If, however, a backset should occur, the patient should receive the third injection and advantage taken of the healing thus obtained, and mercury and potassium iodid continued until the disease is cured.

Ehrlich says that the latest reports indicate that the most satisfactory and permanent results follow the intravenous injections and that he recommends it as being far superior to any other method. Although it is a little more difficult to administer, it is attended with practically no pain and the dose may be repeated when needed without danger, as there is none of the remedy stored and retained, as is sometimes the case in the subcutaneous and intramuscular injections. The inconvenience to the patient when the intravenous method is used is so slight that he can the more easily be induced to take other injections until we have reasonable ground for believing the cure permanent. As yet we have no reason for shortening the time in which a patient may marry with impunity.

A mistaken idea is prevalent among the laity and uninformed physicians that "606" is always a "single shot" remedy. Such we have known for months is not necessarily the case, and if such claims are made for it much harm will be done to patients by only partially curing them and reproach brought upon perhaps our most powerful remedy in combatting syphilis. Let us use more common sense, everyday horse-sense, in both the administration of salvarsan, as well as in the prognosis, and thus not disappoint our patients and ourselves.

E. G. BALLENGER, M. D.

